# Overcoming the challenges in intra-hospital transport of COVID patients

**DOI:** 10.1186/s42077-022-00241-2

**Published:** 2022-05-04

**Authors:** Divya Jain, Ankur Luthra, Neeru Sahni, GoverdhanDuttPuri

**Affiliations:** Anaesthesia and Intensive Care, PGIMER, Sector 12, Chandigarh, PIN 160012 India

To the Editor

When the going gets tough, the tough get going!!

Safe transportation of critically ill patients is always challenging and primarily depends on staff training, pre-defined hospital transport protocols and checklists, the availability of appropriate transport equipment, and the timing of transport (Eiding et al. 2019; Munjal et al. 2020). We, at a tertiary care facility in Northern India, have dealt with the challenges of transporting patients within the organizational division of COVID, non-COVID, and COVID suspect units facilitating appropriate infection control measures to minimize the risk of transmission of the virus. Our designated COVID hospital was organized into medical and surgical wards, intensive care units (ICUs), and operation theatres (OT), catering to both medical and surgical conditions of COVID patients. The immense increase in the number of not just critically ill COVID patients but patients with emergent surgical needs, presenting to our tertiary center called for building up of effective teams and protocols for intra-hospital transport of these patients between wards, emergency department, intensive care unit, and radiology suites.

To ensure seamless transfer of these patients, an action plan was outlined. The primary components included the following:

A Formulation of the checklist
B Training the non-clinical health care workers
C Triaging the patients
D Monitoring and surveillance
  A *Formulation of the checklist*: to minimizing the risk of critical events during the transport process checklists were prepared. These checklists included the pre-transport equipment, drug and communication check prior to each transport were displayed in the triage areas and all the ambulances engaged in shifting of the COVID-19 patients (Yousuf et al. 2020) (Table 1).
  B *Training the non-clinical HCW*: shortage of trained staff was the biggest obstacle as the trained anesthesia staff including the residents and the technical staff was already engaged in the intensive care units and 24-h functional operational theatres. To overcome this, the resident staff working in non-clinical medical branches like anatomy, pharmacology, pathology was deployed. These residents underwent comprehensive training before their duty.

A training schedule curriculum included the following:

a Training on the infection control policies: all members of the transport team were trained in infection control policies like donning and doffing skills.
b Orientation of the COVID hospital: a complete orientation of the COVID hospital and different organizational divisions was provided by the hospital administration department including the designated donning and doffing areas in the COVID hospital (Singh et al. 2020).
c Clinical training included teaching them patient assessment before transport and how to conduct pre-transport equipment check. Emphasis was laid to ensure the stability of patient prior to transport.
d Training on team dynamics. As the transport protocols warrant minimal staff for transport of COVID patients, a 3-member team consisting of a resident, nursing officer, and a hospital attendant (porter) was formulated. Each member of the team was designated specified responsibilities and trained accordingly.
e Hands on demonstration of the equipments: the technique of mask ventilation using a face mask and AMBU bag, endotracheal intubation, use of Bains circuit, or portable ventilator for ventilation was demonstrated.
  A *Triaging the patients*: knowing the limitation of our relatively inexperienced staff, the concept of triaging the transport based on the clinical status of the patient was done. Relatively, stable patients including those requiring transfer to surgical or radiological suites were shifted by the non-clinical residents. The anesthesia team was called for assisting in the shifting very sick patients, including intubated, tracheostomized patients, and patients on high ionotropic support, as these patients had increased risk of witnessing adverse events warranting resuscitation (Bourn et al. 2018).
  B *Monitoring and surveillance*: the shifting of COVID patients was constantly monitored by the control room personnel comprising of both anesthesiology and hospital administration staff using closed WhatsApp group and the status of each transfer was constantly updated by the shifting resident to appraise the whole management team about the safe transfer of any critically or non-critically ill patient The logistics were taken care of by the administration and the technical aspects were monitored by experienced anesthetists in order to reduce time delays as well as minimize any mishaps due to less experienced non-clinical residents who were involved in shifting team.

So far, we have had been successful in implementing this approach and ensuring a smooth intra-hospital transfer of COVID-19 patients. We hope this protocolized approach can be useful for other COVID care centers in strengthening and streamlining the transport process.

## Data Availability

None.

## Abbreviations

AMBU: Artificial manual breathing unit
HCW: Health care workers
